# Supplementary material for: Epigenetic age acceleration, telomere length, and neurocognitive function in long-term survivors of childhood cancer
Source: Nat Commun. 2025 Nov 27;16:10655. doi: 10.1038/s41467-025-65664-5 (PMC12660399; doi:10.1038/s41467-025-65664-5)
Supplement: Supplementary file 1 — Supplementary Information [file 41467_2025_65664_MOESM1_ESM.pdf]

Supplementary Figure 1: Participant enrollment and completion flowchart

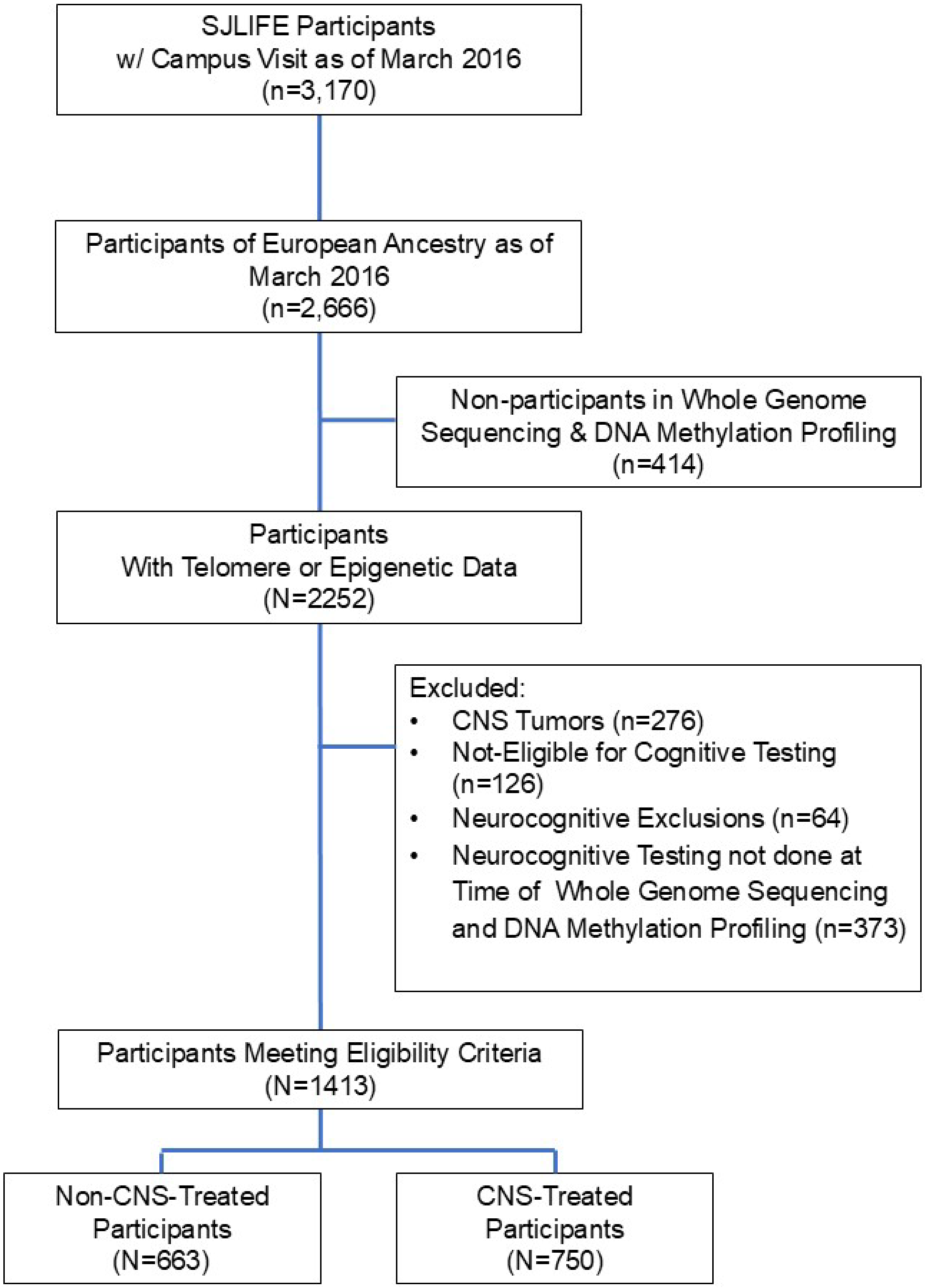

Supplementary Table 1: Characteristics of participants who were and were not eligible for analyses stratified by CNS treatment status.

	Non-CNS-Treated Survivors			CNS-Treated Survivors		
	With Biomarker Data (n=663)	Without Biomarker Data (n=347)	P[1]	With Biomarker Data (n=750)	Without Biomarker Data (n=377)	P[1]
	Mean (SD)	Mean (SD)		Mean (SD)	Mean (SD)	
Age at baseline (years)	34.3 (9.0)	32.4 (8.4)	0.001	32.3 (8.2)	31.2 (7.7)	0.029
Age at diagnosis (years)	9.9 (6.5)	7.8 (6.1)	<0.001	7.3 (4.8)	7.3 (4.9)	0.911
Time from diagnosis to baseline (years)	24.4 (8.9)	24.5 (7.9)	0.834	25.0 (8.0)	24.0 (7.7)	0.033
	N(%)	N(%)		N(%)	N(%)	
Sex						
Male	352 (53.1)	173 (49.9)	0.328	395 (52.7)	200 (53.0)	0.921
Female	311 (46.9)	174 (50.1)		354 (47.3)	177 (47.0)	
Diagnosis						
Acute lymphoblastic leukemia	1 (0.2)	0 (0.0)		577 (76.9)	280 (74.3)	
Acute myeloid leukemia	6 (0.9)	2 (0.6)		39 (5.2)	37 (9.8)	
Ewing sarcoma family of tumors	55 (8.3)	16 (4.6)		3 (0.4)	2 (0.5)	
Hodgkin lymphoma	211 (31.8)	63 (18.2)		5 (0.7)	1 (0.3)	
Neuroblastoma	60 (9.1)	41 (11.8)		0 (0.0)	0 (0.0)	
Non-Hodgkin lymphoma	28 (4.2)	15 (4.3)		105 (14.0)	36 (9.6)	
Osteosarcoma	59 (8.9)	27 (7.8)		2 (0.3)	0 (0.0)	
Other	56 (8.5)	52 (15.0)		8 (1.1)	14 (3.7)	
Retinoblastoma	41 (6.2)	23 (6.6)		0 (0.0)	2 (0.5)	
Rhabdomyosarcoma	41 (6.2)	26 (7.5)		9 (1.2)	3 (0.8)	
Soft tissue sarcoma	32 (4.8)	18 (5.2)		1 (0.1)	2 (0.5)	
Wilms tumor	73 (11.0)	64 (18.4)		1 (0.1)	0 (0.0)	
Radiation						
No radiation treatment	242 (36.5)	168 (48.4)	<0.001	334 (44.5)	135 (35.8)	0.005
Any radiation treatment	421 (63.5)	179 (51.6)		416 (55.5)	242 (64.2)	
Brain radiation (yes)	N/A	N/A		379 (50.5)	232 (61.5)	<0.001
> 0 to 20 Gy	N/A	N/A		121 (31.9)	104 (44.8)	0.006
> 20 to 35 Gy	N/A	N/A		142 (37.7)	70 (30.2)	
> 35 Gy	N/A	N/A		116 (30.6)	58 (25.0)	
Chest (yes)	312 (46.9)	114 (26.7)	<0.001	94 (12.5)	106 (53.3)	<0.001
<=35 Gy	131 (42.0)	57 (50.0)	0.140	74 (79.6)	95 (89.6)	0.048
> 35 Gy	181 (58.0)	57 (50.0)		19 (20.4)	11 (10.4)	
Chemotherapy						
High-dose IV cytarabine (yes)	5 (0.8)	3 (0.9)	0.851	70 (9.3)	72 (19.1)	<0.001
High-dose IV methotrexate (yes)	60 (9.1)	21 (6.1)	0.096	396 (52.8)	196 (52.0)	0.780
Intrathecal methotrexate (yes)	N/A	N/A		697 (92.9)	334 (88.6)	0.014

CNS: Central Nervous System; SD: Standard Deviation; Gy: Gray; IV: Intravenous; N/A: Not Applicable
[1] two-sample t-tests or chi-square tests

Supplementary Table 2: Marginal adjusted means of aging-related biomarkers and mean difference (95%CI) in aging-related biomarkers in survivors compared with controls.

	Controls (n=282)	Non-CNS-Treated (n=663)		CNS-Treated (n=750)		Non-CNS-Treated vs. CNS-Treated	
	Adjusted Mean (95%CI)	Adjusted Mean (95%CI)	Mean Difference[1] (Survivor-Control) (95%CI)	Adjusted Mean (95%CI)	Mean Difference[1] (Survivor-Control) (95%CI)	Mean Difference[1] (non-CNS, CNS-Treated) (95%CI)	p-value
EAA (years)							
Levine's	-3.34 (-3.91, -2.79)	1.30 (0.91, 1.69)	4.64 (3.98, 5.31)	0.36 (0.01, 0.70)	3.69 (3.11, 4.28)	0.88 (0.38, 1.39)	0.001
Horvath	-1.54 (-1.94, -1.14)	0.67 (0.41, 0.93)	2.22 (1.78, 2.67)	-0.18 (-0.46, 0.10)	1.35 (0.88, 1.82)	0.83 (0.49, 1.18)	<0.001
Hannum	-3.81 (-4.39, -3.22)	1.47 (1.08, 1.87)	5.25 (4.58, 5.93)	0.05 (-0.32, 0.43)	3.90 (3.27, 4.53)	1.22 (0.69, 1.74)	<0.001
PCGrimAge	-0.84 (-1.12, -0.57)	0.84 (0.65, 1.03)	1.63 (1.31, 1.95)	0.72 (0.55, 0.89)	1.58 (1.29, 1.87)	0.04 (-0.20, 0.29)	0.732
Dunedin PACE	0.78 (0.77, 0.79)	0.85 (0.85, 0.86)	0.07 (0.06, 0.09)	0.83 (0.82, 0.84)	0.05 (0.04, 0.06)	0.02 (0.01, 0.03)	<0.001
mLTL (kb)							
Telomere length	6.47 (6.33, 6.62)	6.01 (5.92, 6.11)	-0.47 (-0.64, -0.31)	6.15 (6.05, 6.25)	-0.30 (-0.47, -0.13)	-0.17 (-0.30, -0.04)	0.011
Telomere residual	0.33 (0.19, 0.47)	-0.10 (-0.19, -0.01)	-0.46 (-0.61, -0.30)	-0.08 (-0.18, 0.01)	-0.39 (-0.55, -0.23)	-0.06 (-0.18, 0.06)	0.340

CNS: Central Nervous System; CI: Confidence Interval; EAA: Epigenetic Age Acceleration; mLTL: mean Leukocyte Telomere Length
[1]Linear regression adjusted for sex, BMI, smoking, and physical activity. All survivor vs. control comparisons were two sided, t-tests from linear regression, and statistically significantly different at p<0.001.

Supplementary Table 3: St. Jude Lifetime cohort neurocognitive battery.

Cognitive Domain	Neuropsychological Assessment
Global Cognition	
Verbal reasoning	Wechsler Abbreviated Scale of Intelligence -II Vocabulary[1]
Non-verbal reasoning	Wechsler Abbreviated Scale of Intelligence -II Matrix Reasoning[1]
Academics	
Word reading	Woodcock Johnson-III Letter-Word Identification [2]
Mathematics	Woodcock Johnson-III Calculation [2]
Attention	
Sustained	Conner's Continuous Performance Task Omissions[3]
Variability	Conner's Continuous Performance Task Variability[3]
Commissions	Conner's Continuous Performance Task Commissions[3]
Focused	Trail Making Test A[4]
Processing speed	
Visual-motor	Wechsler Abbreviated Scale of Intelligence Coding[1]
Motor	Grooved Peg Board[5]
Memory	
Span	Wechsler Adult Intelligence Scale-III/IV Digit Span Forward[6]
New verbal encoding	California Verbal Learning Test Trials 1-5[7]
Short-term verbal recall	California Verbal Learning Test Short-Term Free Recall[7]
Long-term verbal recall	California Verbal Learning Test Long-Term Free Recall [7]
Executive function	
Perseveration	Conner's Continuous Performance Task Perseveration[3]
Working memory	Wechsler Adult Intelligence Scale-III/IV Digit Span Backward[6]
Cognitive switching/flexibility	Trail Making Test B[4]
Verbal fluency	Verbal Fluency (FAS)[8]

1. Wechsler D. *Abbreviated Scale of Intelligence*. San Antonio, TX: Pyschological Corporation; 1999.
2. Woodcock RW, McGrew, K.S., Mather, N. *Woodcock-Johnson III Tests of Acheivement*. Itasca, IL: Riverside Publishing; 2001.
3. Conners CK, Connelly, V., Campbell, S., MacLean, M. *Conners' Continuous Performance Test, 2nd ed.* North Tonawanda, NY: Multi-Health Systems Inc. ; 2003.
4. Tombaugh TN. Trail Making Test A and B: normative data stratified by age and education. Arch Clin Neuropsychol 2004;19(2):203-14.
5. Strauss E, Sherman, E.M., Spreen,). *A Compendium of Neuropsychological Tests: Adminstration, Norms, and Commentary, 3rd ed.* . London, UK: Oxford University Press; 2006.
6. Wechsler D. *Wechsler Adult Intelligence Scale, 4th ed.* San Antonio, TX: Pyschological Corporation; 2008.
7. Delis DC, Kramer, J.H., Kaplan, E. *California Verbal Learning Test, 2nd ed.* San Antonio, TX; 2000.
8. Delis DC, Kaplan, E., Kramer, J.H. *Delis-Kaplan Executive Function System*. San Antonio, TX: Psychological Corporation; 2001.

Supplementary Table 4: Mean difference (95%CI) in neurocognitive z-score associated with the second or third tertile of PCPhenoAge EAA compared to the first tertile stratified by exposure to CNS-directed therapy and further adjusted for education.

	Non-CNS-Treated[1] n=663				CNS-Treated[2] n=750			
	Tertile 2 vs. 1		Tertile 3 vs. 1		Tertile 2 vs. 1		Tertile 3 vs. 1	
	β(95%CI)	p[3]	β(95%CI)	p[3]	β(95%CI)	p[3]	β(95%CI)	p[3]
Global Cognition								
Verbal reasoning	0.03(-0.16,0.22)	0.948	-0.07(-0.26,0.12)	0.459	-0.07(-0.24,0.09)	0.642	-0.10(-0.28,0.08)	0.478
Non-verbal reasoning	-0.01(-0.16,0.15)	0.948	-0.17(-0.32,-0.02)	0.088	-0.03(-0.18,0.13)	0.733	-0.08(-0.24,0.09)	0.478
Academics								
Word reading	0.00(-0.10,0.11)	0.948	-0.06(-0.16,0.04)	0.299	-0.07(-0.18,0.03)	0.642	-0.10(-0.21,0.02)	0.361
Mathematics	-0.13(-0.29,0.03)	0.487	-0.16(-0.32,0.00)	0.106	-0.06(-0.22,0.10)	0.642	-0.06(-0.23,0.11)	0.503
Attention								
Sustained	0.15(-0.07,0.37)	0.310	-0.13(-0.35,0.09)	0.497	0.01(-0.21,0.24)	0.948	-0.06(-0.30,0.18)	0.645
Variability	0.04(-0.19,0.26)	0.748	-0.14(-0.36,0.07)	0.497	-0.06(-0.26,0.14)	0.948	-0.10(-0.31,0.12)	0.645
Commissions	0.12(-0.08,0.33)	0.310	-0.04(-0.24,0.16)	0.693	0.06(-0.13,0.25)	0.948	-0.05(-0.26,0.16)	0.645
Focused attention	-0.15(-0.35,0.05)	0.310	-0.08(-0.28,0.12)	0.562	-0.01(-0.21,0.20)	0.948	-0.06(-0.27,0.16)	0.645
Processing Speed								
Visual-motor	-0.01(-0.18,0.16)	0.933	-0.17(-0.34,-0.00)	0.092	-0.04(-0.21,0.13)	0.846	-0.02(-0.20,0.15)	0.785
Motor	0.07(-0.15,0.29)	0.933	0.04(-0.18,0.25)	0.732	0.02(-0.19,0.23)	0.846	0.04(-0.18,0.26)	0.785
Memory								
Span	0.15(-0.04,0.34)	0.501	-0.11(-0.30,0.07)	0.937	-0.03(-0.20,0.15)	0.768	-0.02(-0.20,0.17)	0.977
New verbal encoding	0.08(-0.13,0.29)	0.709	-0.01(-0.21,0.20)	0.937	-0.10(-0.30,0.10)	0.546	-0.08(-0.29,0.14)	0.976
Short-term verbal recall	-0.06(-0.26,0.14)	0.709	-0.06(-0.26,0.14)	0.937	-0.08(-0.28,0.11)	0.546	-0.003(-0.21,0.21)	0.977
Long-term verbal recall	0.04(-0.17,0.25)	0.709	0.02(-0.18,0.23)	0.937	-0.14(-0.34,0.07)	0.546	-0.08(-0.31,0.14)	0.976
Executive Function								
Perseveration	0.17(-0.07,0.40)	0.705	-0.09(-0.32,0.15)	0.467	0.04(-0.21,0.29)	0.764	0.09(-0.17,0.36)	0.667
Working memory	0.08(-0.09,0.26)	0.717	-0.10(-0.27,0.07)	0.467	0.06(-0.10,0.22)	0.764	0.002(-0.17,0.17)	0.980
Cognitive switching/flexibility	-0.01(-0.26,0.24)	0.951	-0.16(-0.40,0.09)	0.467	-0.10(-0.35,0.15)	0.764	-0.16(-0.43,0.10)	0.667
Verbal fluency	0.02(-0.20,0.23)	0.951	-0.10(-0.31,0.12)	0.467	-0.03(-0.21,0.15)	0.764	-0.07(-0.26,0.12)	0.667

EAA: Epigenetic Age Acceleration calculated used PCPhenoAge epigenetic clock.

[1]Models are adjusted for sex, diagnosis age, education, BMI, physical activity, smoking, and high-dose IV methotrexate, [2]Models are adjusted for sex, diagnosis age, education, BMI, physical activity, smoking, cranial radiation dose, intrathecal methotrexate, high-dose IV methotrexate, high-dose cytarabine, and neurosurgery, [3] p-values from linear regression models' t-tests, which are two sided, and adjusted for multiple comparisons using the false-discovery rate

Supplementary Table 5: Mean difference (95%CI) in neurocognitive z-score associated with the second or third tertile of PCGrimAge EAA compared to the first tertile stratified by exposure to CNS-directed therapy and further adjusted for education.

	Non-CNS-Treated[1] n=663				CNS-Treated[2] n=750			
	Tertile 2 vs. 1		Tertile 3 vs. 1		Tertile 2 vs. 1		Tertile 3 vs. 1	
	β(95%CI)	p[3]	β(95%CI)	p[3]	β(95%CI)	p[3]	β(95%CI)	p[3]
Global Cognition								
Verbal reasoning	-0.02(-0.21,0.16)	0.919	-0.25(-0.45,-0.05)	0.020	-0.27(-0.43,-0.10)	0.008	-0.35(-0.54,-0.16)	0.001
Non-verbal reasoning	-0.01(-0.16,0.14)	0.919	-0.19(-0.35,-0.03)	0.020	-0.18(-0.34,-0.02)	0.055	-0.16(-0.34,0.02)	0.081
Academics								
Word reading	-0.06(-0.16,0.04)	0.908	-0.15(-0.26,-0.05)	0.011	-0.09(-0.20,0.01)	0.114	-0.19(-0.31,-0.07)	0.005
Mathematics	-0.06(-0.22,0.10)	0.908	-0.25(-0.42,-0.07)	0.011	-0.12(-0.29,0.04)	0.152	-0.18(-0.37,0.01)	0.078
Attention								
Sustained	-0.06(-0.28,0.16)	0.704	-0.18(-0.41,0.06)	0.181	-0.25(-0.48,-0.03)	0.058	-0.27(-0.53,-0.01)	0.082
Variability	-0.11(-0.33,0.11)	0.704	-0.28(-0.51,-0.04)	0.081	-0.21(-0.41,-0.00)	0.066	-0.36(-0.60,-0.13)	0.010
Commissions	0.04(-0.16,0.24)	0.704	0.06(-0.16,0.28)	0.586	-0.15(-0.35,0.05)	0.139	-0.06(-0.29,0.16)	0.590
Focused attention	-0.08(-0.28,0.11)	0.704	-0.20(-0.41,0.01)	0.117	-0.24(-0.45,-0.03)	0.058	-0.19(-0.43,0.04)	0.143
Processing Speed								
Visual-motor	-0.07(-0.24,0.10)	0.739	-0.28(-0.46,-0.10)	0.005	-0.26(-0.43,-0.09)	0.005	-0.28(-0.47,-0.08)	0.010
Motor	0.04(-0.18,0.26)	0.739	-0.11(-0.34,0.12)	0.361	-0.28(-0.49,-0.07)	0.009	-0.27(-0.51,-0.03)	0.030
Memory								
Span	-0.18(-0.36,0.01)	0.251	-0.24(-0.44,-0.04)	0.080	-0.19(-0.36,-0.01)	0.054	-0.18(-0.38,0.03)	0.116
New verbal encoding	0.16(-0.05,0.37)	0.251	-0.14(-0.36,0.08)	0.222	-0.28(-0.48,-0.07)	0.033	-0.21(-0.45,0.02)	0.116
Short-term verbal recall	-0.07(-0.28,0.13)	0.475	-0.17(-0.38,0.05)	0.222	-0.11(-0.31,0.09)	0.291	-0.10(-0.33,0.12)	0.375
Long-term verbal recall	0.11(-0.10,0.32)	0.390	-0.15(-0.37,0.07)	0.222	-0.24(-0.45,-0.03)	0.053	-0.28(-0.52,-0.03)	0.107
Executive Function								
Perseveration	0.10(-0.14,0.34)	0.546	-0.09(-0.35,0.16)	0.480	0.04(-0.22,0.29)	0.777	-0.14(-0.43,0.16)	0.362
Working memory	-0.13(-0.31,0.04)	0.264	-0.25(-0.43,-0.06)	0.034	-0.19(-0.36,-0.03)	0.044	-0.18(-0.37,0.00)	0.106
Cognitive switching/flexibility	0.08(-0.17,0.32)	0.546	-0.19(-0.45,0.07)	0.200	-0.21(-0.46,0.04)	0.139	-0.19(-0.47,0.10)	0.269
Verbal fluency	-0.19(-0.40,0.03)	0.264	-0.27(-0.50,-0.04)	0.038	-0.21(-0.39,-0.03)	0.044	-0.22(-0.42,-0.01)	0.106

EAA: Epigenetic Age Acceleration calculated used PCGrimAge epigenetic clock.

[1]Models are adjusted for sex, diagnosis age, education, BMI, physical activity, smoking, and high-dose IV methotrexate, [2]Models are adjusted for sex, diagnosis age, education, BMI, physical activity, smoking, cranial radiation dose, intrathecal methotrexate, high-dose IV methotrexate, high-dose cytarabine, and neurosurgery, [3] p-values from linear regression models' t-tests, which are two sided, and adjusted for multiple comparisons using the false-discovery rate

Supplementary Table 6: Mean difference (95%CI) in neurocognitive z-score associated with the second or third tertile of **DunedinPACE** to the first tertile stratified by exposure to CNS-directed therapy and further adjusted for education.

	Non-CNS-Treated[1] n=663				CNS-Treated[2] n=750			
	Tertile 2 vs. 1		Tertile 3 vs. 1		Tertile 2 vs. 1		Tertile 3 vs. 1	
	β(95%CI)	p[3]	β(95%CI)	p[3]	β(95%CI)	p[3]	β(95%CI)	p[3]
Global Cognition								
Verbal reasoning	-0.11(-0.30,0.09)	0.544	-0.22(-0.42,-0.02)	0.057	-0.12(-0.29,0.05)	0.237	-0.20(-0.39,-0.02)	0.058
Non-verbal reasoning	-0.01(-0.17,0.14)	0.887	-0.09(-0.25,0.07)	0.272	-0.13(-0.29,0.03)	0.237	-0.20(-0.37,-0.02)	0.058
Academics								
Word reading	-0.06(-0.17,0.04)	0.544	-0.12(-0.23,-0.01)	0.057	-0.04(-0.15,0.06)	0.442	-0.11(-0.23,0.01)	0.065
Mathematics	-0.04(-0.21,0.13)	0.850	-0.17(-0.34,0.01)	0.077	-0.11(-0.28,0.05)	0.237	-0.19(-0.38,-0.01)	0.058
Attention								
Sustained	0.19(-0.04,0.41)	0.197	-0.001(-0.24,0.23)	0.992	0.06(-0.17,0.28)	0.641	-0.29(-0.54,-0.03)	0.045
Variability	-0.07(-0.29,0.16)	0.565	-0.27(-0.50,-0.03)	0.102	-0.11(-0.32,0.10)	0.594	-0.27(-0.50,-0.04)	0.045
Commissions	0.19(-0.02,0.40)	0.197	0.14(-0.08,0.36)	0.411	0.05(-0.15,0.24)	0.641	-0.03(-0.25,0.19)	0.779
Focused attention	0.15(-0.05,0.36)	0.197	-0.09(-0.30,0.13)	0.570	0.13(-0.08,0.33)	0.594	-0.25(-0.48,-0.02)	0.045
Processing Speed								
Visual-motor	0.14(-0.04,0.31)	0.130	-0.08(-0.26,0.10)	0.462	-0.04(-0.21,0.13)	0.627	-0.23(-0.42,-0.04)	0.031
Motor	0.22(-0.01,0.44)	0.121	-0.09(-0.32,0.14)	0.462	0.07(-0.14,0.28)	0.627	-0.19(-0.43,0.04)	0.108
Memory								
Span	0.07(-0.12,0.26)	0.481	-0.19(-0.39,0.01)	0.278	-0.02(-0.20,0.15)	0.796	-0.02(-0.22,0.18)	0.838
New verbal encoding	0.20(-0.02,0.42)	0.274	0.06(-0.16,0.28)	0.577	-0.36(-0.56,-0.15)	0.001	-0.22(-0.44,0.01)	0.127
Short-term verbal recall	0.12(-0.09,0.33)	0.362	0.08(-0.13,0.30)	0.577	-0.26(-0.46,-0.06)	0.013	-0.17(-0.39,0.06)	0.188
Long-term verbal recall	0.13(-0.08,0.34)	0.362	0.12(-0.10,0.34)	0.577	-0.40(-0.61,-0.19)	0.001	-0.31(-0.55,-0.08)	0.037
Executive Function								
Perseveration	0.17(-0.08,0.41)	0.247	-0.11(-0.36,0.14)	0.810	-0.11(-0.37,0.14)	0.503	-0.28(-0.56,0.00)	0.104
Working memory	0.10(-0.08,0.28)	0.293	-0.08(-0.26,0.11)	0.810	-0.004(-0.17,0.16)	0.959	-0.03(-0.21,0.15)	0.746
Cognitive switching/flexibility	0.32(0.06,0.57)	0.056	-0.03(-0.29,0.23)	0.810	0.13(-0.12,0.38)	0.503	-0.37(-0.65,-0.09)	0.036
Verbal fluency	0.18(-0.05,0.40)	0.247	-0.05(-0.28,0.18)	0.810	-0.11(-0.29,0.07)	0.503	-0.12(-0.33,0.08)	0.314

EAA: Epigenetic Age Acceleration calculated used DunedinPACE epigenetic clock.

[1]Models are adjusted for sex, diagnosis age, education, BMI, physical activity, smoking, and high-dose IV methotrexate, [2]Models are adjusted for sex, diagnosis age, education, BMI, physical activity, smoking, cranial radiation dose, intrathecal methotrexate, high-dose IV methotrexate, high-dose cytarabine, and neurosurgery, [3] p-values from linear regression models' t-tests, which are two sided, and adjusted for multiple comparisons using the false-discovery rate

Supplementary Table 7: Mean difference (95%CI) in neurocognitive z-score associated with the second or third tertile of **Hannum** EAA compared to the first tertile stratified by exposure to CNS-directed therapy.

	Non-CNS-Treated[1] n=663				CNS-Treated[2] n=750			
	Tertile 2 vs. 1		Tertile 3 vs. 1		Tertile 2 vs. 1		Tertile 3 vs. 1	
	β(95%CI)	p[3]	β(95%CI)	p[3]	β(95%CI)	p[3]	β(95%CI)	p[3]
Global Cognition								
Verbal reasoning	-0.13(-0.34,0.07)	0.319	-0.15(-0.35,0.04)	0.128	-0.19(-0.38,-0.01)	0.086	-0.19(-0.39,0.01)	0.128
Non-verbal reasoning	0.001(-0.15,0.15)	0.993	-0.15(-0.30,-0.00)	0.085	-0.17(-0.33,-0.01)	0.086	-0.12(-0.29,0.06)	0.200
Academics								
Word reading	-0.07(-0.18,0.04)	0.319	-0.15(-0.26,-0.04)	0.028	-0.10(-0.21,0.01)	0.101	-0.12(-0.24,0.00)	0.128
Mathematics	-0.15(-0.33,0.02)	0.319	-0.13(-0.30,0.04)	0.128	-0.10(-0.28,0.07)	0.247	-0.12(-0.31,0.07)	0.200
Attention								
Sustained	0.01(-0.21,0.22)	0.951	-0.12(-0.33,0.09)	0.498	-0.21(-0.43,0.00)	0.119	-0.08(-0.31,0.16)	0.886
Variability	-0.08(-0.30,0.14)	0.951	-0.21(-0.42,0.00)	0.208	-0.19(-0.38,0.01)	0.119	-0.12(-0.33,0.09)	0.886
Commissions	0.01(-0.19,0.21)	0.951	-0.03(-0.22,0.16)	0.955	0.08(-0.11,0.26)	0.434	-0.01(-0.22,0.19)	0.891
Focused attention	0.07(-0.13,0.26)	0.951	-0.01(-0.19,0.18)	0.955	-0.08(-0.29,0.12)	0.434	-0.05(-0.27,0.17)	0.886
Processing Speed								
Visual-motor	-0.11(-0.29,0.06)	0.412	-0.17(-0.34,-0.01)	0.087	-0.13(-0.30,0.04)	0.254	-0.08(-0.26,0.11)	0.403
Motor	0.002(-0.21,0.22)	0.986	-0.001(-0.21,0.21)	0.990	-0.12(-0.32,0.08)	0.254	-0.20(-0.42,0.02)	0.139
Memory								
Span	0.05(-0.14,0.24)	0.606	-0.06(-0.25,0.12)	0.875	-0.04(-0.21,0.13)	0.617	-0.15(-0.33,0.03)	0.318
New verbal encoding	-0.11(-0.32,0.10)	0.575	-0.05(-0.25,0.15)	0.875	-0.19(-0.39,0.01)	0.180	-0.15(-0.37,0.07)	0.318
Short-term verbal recall	-0.13(-0.33,0.07)	0.575	-0.09(-0.28,0.10)	0.875	-0.17(-0.36,0.03)	0.180	-0.12(-0.33,0.09)	0.318
Long-term verbal recall	-0.08(-0.29,0.12)	0.587	-0.01(-0.20,0.19)	0.957	-0.13(-0.33,0.08)	0.309	-0.11(-0.34,0.11)	0.318
Executive Function								
Perseveration	-0.06(-0.30,0.17)	0.980	-0.25(-0.48,-0.03)	0.111	-0.17(-0.40,0.07)	0.340	-0.09(-0.35,0.16)	0.619
Working memory	0.002(-0.18,0.18)	0.980	-0.04(-0.21,0.14)	0.683	-0.07(-0.23,0.09)	0.399	-0.04(-0.21,0.13)	0.619
Cognitive switching/flexibility	-0.03(-0.28,0.23)	0.980	-0.10(-0.34,0.14)	0.566	-0.15(-0.41,0.11)	0.341	-0.25(-0.53,0.03)	0.163
Verbal fluency	-0.18(-0.40,0.04)	0.414	-0.09(-0.30,0.12)	0.566	-0.18(-0.36,0.00)	0.221	-0.17(-0.37,0.02)	0.163

EAA: Epigenetic Age Acceleration calculated used Hannum epigenetic clock.
[1]Models are adjusted for sex, diagnosis age, BMI, physical activity, smoking, and high-dose IV methotrexate, [2]Models are adjusted for sex, diagnosis age, BMI, physical activity, smoking, cranial radiation dose, intrathecal methotrexate, high-dose IV methotrexate, high-dose cytarabine, and neurosurgery, [3] p-values from linear regression models' t-tests, which are two sided, and adjusted for multiple comparisons using the false-discovery rate

Supplementary Table 8: Mean difference (95%CI) in neurocognitive z-score associated with the second or third tertile of **Horvath** EAA compared to the first tertile stratified by exposure to CNS-directed therapy.

	Non-CNS-Treated[1] n=663				CNS-Treated[2] n=750			
	Tertile 2 vs. 1		Tertile 3 vs. 1		Tertile 2 vs. 1		Tertile 3 vs. 1	
	β(95%CI)	p[3]	β(95%CI)	p[3]	β(95%CI)	p[3]	β(95%CI)	p[3]
Global Cognition								
Verbal reasoning	-0.11(-0.31,0.09)	0.569	-0.04(-0.24,0.16)	0.696	-0.02(-0.21,0.16)	0.923	-0.000(-0.19,0.19)	0.997
Non-verbal reasoning	0.02(-0.13,0.17)	0.811	-0.05(-0.19,0.10)	0.696	0.14(-0.02,0.30)	0.382	0.09(-0.07,0.26)	0.997
Academics								
Word reading	0.03(-0.08,0.14)	0.745	-0.02(-0.13,0.09)	0.696	-0.01(-0.12,0.11)	0.923	0.01(-0.11,0.12)	0.997
Mathematics	0.14(-0.03,0.31)	0.409	0.08(-0.09,0.25)	0.696	-0.01(-0.19,0.17)	0.923	-0.06(-0.24,0.13)	0.997
Attention								
Sustained	-0.12(-0.33,0.09)	0.775	0.08(-0.13,0.29)	0.715	0.06(-0.17,0.28)	0.932	0.01(-0.21,0.24)	0.897
Variability	-0.08(-0.29,0.13)	0.775	0.15(-0.06,0.36)	0.674	0.02(-0.18,0.22)	0.932	0.05(-0.16,0.25)	0.897
Commissions	-0.06(-0.25,0.14)	0.775	-0.04(-0.23,0.16)	0.715	-0.01(-0.20,0.18)	0.932	-0.05(-0.24,0.14)	0.897
Focused attention	-0.02(-0.21,0.17)	0.823	0.04(-0.15,0.23)	0.715	-0.01(-0.22,0.20)	0.932	0.03(-0.18,0.24)	0.897
Processing Speed								
Visual-motor	0.02(-0.15,0.19)	0.968	0.08(-0.09,0.25)	0.702	0.06(-0.11,0.24)	0.559	-0.001(-0.18,0.18)	0.989
Motor	0.004(-0.21,0.22)	0.968	0.04(-0.17,0.25)	0.705	0.06(-0.14,0.27)	0.559	0.01(-0.20,0.22)	0.989
Memory								
Span	0.01(-0.18,0.19)	0.943	-0.09(-0.28,0.09)	0.794	0.12(-0.05,0.29)	0.617	0.05(-0.13,0.22)	0.590
New verbal encoding	-0.07(-0.27,0.14)	0.692	0.03(-0.18,0.23)	0.794	-0.09(-0.29,0.12)	0.617	-0.10(-0.31,0.11)	0.590
Short-term verbal recall	-0.08(-0.27,0.12)	0.692	0.05(-0.14,0.25)	0.794	-0.07(-0.27,0.12)	0.617	-0.07(-0.27,0.13)	0.590
Long-term verbal recall	-0.09(-0.29,0.12)	0.692	0.03(-0.17,0.23)	0.794	-0.04(-0.25,0.17)	0.727	-0.07(-0.28,0.14)	0.590
Executive Function								
Perseveration	-0.12(-0.35,0.11)	0.568	-0.02(-0.25,0.21)	0.853	-0.07(-0.31,0.18)	0.593	0.11(-0.14,0.36)	0.390
Working memory	0.08(-0.10,0.25)	0.568	0.02(-0.16,0.19)	0.853	0.11(-0.05,0.27)	0.312	0.11(-0.05,0.28)	0.336
Cognitive switching/flexibility	0.10(-0.15,0.35)	0.568	0.16(-0.09,0.40)	0.829	0.16(-0.10,0.43)	0.312	0.12(-0.15,0.39)	0.390
Verbal fluency	-0.03(-0.24,0.19)	0.815	-0.05(-0.26,0.16)	0.853	-0.11(-0.29,0.07)	0.312	-0.23(-0.42,-0.04)	0.068

EAA: Epigenetic Age Acceleration calculated used Horvath epigenetic clock.
[1]Models are adjusted for sex, diagnosis age, BMI, physical activity, smoking, and high-dose IV methotrexate, [2]Models are adjusted for sex, diagnosis age, BMI, physical activity, smoking, cranial radiation dose, intrathecal methotrexate, high-dose IV methotrexate, high-dose cytarabine, and neurosurgery, [3] p-values from linear regression models' t-tests, which are two sided, and adjusted for multiple comparisons using the false-discovery rate

Supplementary Table 9: Mean difference (95%CI) in neurocognitive z-score associated with the second or third tertile of **mLTL residual** compared to the first tertile stratified by exposure to CNS-directed therapy.

	Non-CNS-Treated[1] n=663				CNS-Treated[2] n=750			
	Tertile 2 vs. 1		Tertile 3 vs. 1		Tertile 2 vs. 1		Tertile 3 vs. 1	
	β(95%CI)	p[3]	β(95%CI)	p[3]	β(95%CI)	p[3]	β(95%CI)	p[3]
Global Cognition								
Verbal reasoning	0.01(-0.19,0.20)	0.954	0.16(-0.04,0.36)	0.116	0.12(-0.08,0.31)	0.598	0.19(0.00,0.37)	0.196
Non-verbal reasoning	0.04(-0.10,0.18)	0.854	0.12(-0.02,0.27)	0.116	-0.05(-0.22,0.12)	0.722	-0.00(-0.17,0.16)	0.967
Academics								
Word reading	0.03(-0.08,0.13)	0.854	0.15(0.04,0.25)	0.018	0.02(-0.10,0.14)	0.761	0.06(-0.06,0.17)	0.453
Mathematics	0.04(-0.12,0.20)	0.854	0.22(0.06,0.39)	0.018	0.10(-0.09,0.28)	0.598	0.12(-0.05,0.30)	0.329
Attention								
Sustained	-0.13(-0.33,0.07)	0.419	0.05(-0.16,0.26)	0.714	-0.02(-0.25,0.20)	0.994	0.00(-0.22,0.22)	0.999
Variability	-0.19(-0.39,0.02)	0.283	0.08(-0.14,0.29)	0.714	-0.00(-0.21,0.20)	0.994	-0.07(-0.27,0.13)	0.865
Commissions	0.06(-0.13,0.24)	0.566	0.06(-0.13,0.26)	0.714	-0.08(-0.28,0.12)	0.994	-0.04(-0.23,0.14)	0.865
Focused attention	0.09(-0.09,0.28)	0.419	-0.04(-0.23,0.16)	0.714	-0.06(-0.27,0.16)	0.994	-0.05(-0.25,0.16)	0.865
Processing Speed								
Visual-motor	0.01(-0.15,0.18)	0.867	0.09(-0.08,0.26)	0.432	0.02(-0.16,0.20)	0.845	0.07(-0.10,0.25)	0.395
Motor	0.17(-0.03,0.37)	0.18	0.08(-0.13,0.29)	0.432	-0.05(-0.27,0.16)	0.845	-0.16(-0.36,0.05)	0.268
Memory								
Span	0.08(-0.10,0.26)	0.942	0.11(-0.07,0.30)	0.469	-0.02(-0.20,0.16)	0.995	0.02(-0.15,0.19)	0.837
New verbal encoding	0.07(-0.12,0.27)	0.942	0.12(-0.08,0.33)	0.469	0.00(-0.21,0.21)	0.995	-0.06(-0.27,0.14)	0.740
Short-term verbal recall	0.00(-0.18,0.19)	0.966	0.08(-0.12,0.27)	0.480	-0.03(-0.23,0.17)	0.995	-0.11(-0.30,0.09)	0.740
Long-term verbal recall	-0.02(-0.21,0.18)	0.966	0.07(-0.13,0.27)	0.480	-0.01(-0.22,0.21)	0.995	-0.09(-0.29,0.12)	0.740
Executive Function								
Perseveration	-0.05(-0.27,0.17)	0.652	0.03(-0.20,0.26)	0.786	0.15(-0.10,0.40)	0.627	0.10(-0.15,0.34)	0.588
Working memory	0.11(-0.06,0.27)	0.434	0.03(-0.15,0.20)	0.786	0.00(-0.16,0.17)	0.953	0.08(-0.08,0.24)	0.588
Cognitive switching/flexibility	0.06(-0.18,0.30)	0.652	0.06(-0.19,0.30)	0.786	-0.14(-0.41,0.13)	0.627	-0.03(-0.29,0.23)	0.838
Verbal fluency	0.17(-0.04,0.37)	0.434	0.11(-0.10,0.32)	0.786	0.01(-0.18,0.20)	0.953	0.07(-0.11,0.25)	0.588

mLTL: mean leukocyte telomere length residual.

[1]Models are adjusted for sex, diagnosis age, BMI, physical activity, smoking, and high-dose IV methotrexate, [2]Models are adjusted for sex, diagnosis age, BMI, physical activity, smoking, cranial radiation dose, intrathecal methotrexate, high-dose IV methotrexate, high-dose cytarabine, and neurosurgery, [3] p-values from linear regression models' t-tests, which are two sided, and adjusted for multiple comparisons using the false-discovery rate

Supplementary Table 10: Mean difference (95%CI) in neurocognitive z-score associated with the second or third tertile of PCPhenoAge EAA or mLTL residual compared to the first tertile among **survivors of Hodgkin Lymphoma (n=216)**.

	EAA[1]				mLTL[1]			
	Tertile 2 vs. 1		Tertile 3 vs. 1		Tertile 2 vs. 1		Tertile 3 vs. 1	
	β(95%CI)	p[2]	β(95%CI)	p[2]	β(95%CI)	p[2]	β(95%CI)	p[2]
Global Cognition								
Verbal reasoning	-0.15(-0.65,0.36)	0.892	-0.57(-1.04,-0.09)	0.075	-0.09(-0.41,0.23)	0.586	0.06(-0.27,0.40)	0.723
Non-verbal reasoning	-0.08(-0.47,0.31)	0.892	-0.32(-0.68,0.05)	0.119	-0.13(-0.37,0.10)	0.535	-0.06(-0.31,0.19)	0.723
Academics								
Word reading	-0.03(-0.31,0.25)	0.892	-0.26(-0.52,0.01)	0.112	-0.05(-0.22,0.12)	0.586	0.08(-0.11,0.26)	0.723
Mathematics	0.03(-0.38,0.44)	0.892	-0.11(-0.50,0.28)	0.578	-0.17(-0.42,0.07)	0.535	0.09(-0.17,0.35)	0.723
Attention								
Sustained	0.44(-0.15,1.03)	0.399	0.20(-0.36,0.76)	0.894	-0.22(-0.57,0.13)	0.609	-0.15(-0.52,0.23)	0.775
Variability	0.26(-0.29,0.82)	0.472	0.09(-0.44,0.61)	0.894	-0.15(-0.48,0.18)	0.609	-0.19(-0.54,0.16)	0.775
Commissions	0.12(-0.43,0.66)	0.679	0.04(-0.48,0.55)	0.894	-0.01(-0.33,0.32)	0.972	-0.05(-0.39,0.29)	0.775
Focused attention	0.28(-0.15,0.72)	0.399	0.23(-0.18,0.64)	0.894	-0.10(-0.37,0.16)	0.609	-0.04(-0.33,0.24)	0.775
Processing Speed								
Visual-motor	0.33(-0.13,0.79)	0.311	-0.03(-0.47,0.40)	0.909	0.00(-0.28,0.29)	0.993	0.13(-0.18,0.43)	0.420
Motor	-0.01(-0.53,0.51)	0.972	-0.03(-0.52,0.46)	0.909	0.12(-0.19,0.44)	0.878	0.19(-0.14,0.53)	0.420
Memory								
Span	-0.19(-0.69,0.31)	0.446	-0.58(-1.05,-0.11)	0.063	-0.03(-0.35,0.28)	0.950	-0.06(-0.39,0.27)	0.895
New verbal encoding	0.28(-0.24,0.81)	0.446	-0.15(-0.65,0.35)	0.754	0.07(-0.25,0.40)	0.950	0.20(-0.14,0.54)	0.895
Short-term verbal recall	0.39(-0.15,0.93)	0.446	0.11(-0.40,0.62)	0.754	0.01(-0.32,0.34)	0.950	0.11(-0.23,0.46)	0.895
Long-term verbal recall	0.24(-0.30,0.78)	0.446	-0.08(-0.59,0.42)	0.754	-0.01(-0.34,0.32)	0.950	-0.02(-0.37,0.32)	0.895
Executive Function								
Perseveration	0.70(0.05,1.34)	0.134	0.44(-0.17,1.05)	0.264	-0.05(-0.43,0.34)	0.814	-0.13(-0.54,0.28)	0.726
Working memory	-0.31(-0.76,0.15)	0.370	-0.39(-0.82,0.04)	0.264	-0.04(-0.31,0.24)	0.814	-0.13(-0.42,0.17)	0.726
Cognitive switching/flexibility	0.14(-0.43,0.71)	0.635	-0.17(-0.71,0.37)	0.536	-0.11(-0.46,0.24)	0.814	0.04(-0.34,0.41)	0.853
Verbal fluency	-0.14(-0.71,0.42)	0.635	-0.35(-0.88,0.18)	0.264	0.17(-0.18,0.52)	0.814	0.25(-0.12,0.62)	0.713

[1]Models are adjusted for sex, diagnosis age, BMI, physical activity, smoking, and high-dose IV methotrexate, [2] p-values from linear regression models' t-tests, which are two sided, and adjusted for multiple comparisons using the false-discovery rate

Supplementary Table 11: Mean difference (95%CI) in neurocognitive z-score associated with the tertile of either DunedinPACE or PCGrimAge EAA compared to the first tertile among **survivors of Hodgkin Lymphoma (n=216)**.

	Dunedin PACE[1]				PCGrimAge EAA[1]			
	Tertile 2 vs. 1		Tertile 3 vs. 1		Tertile 2 vs. 1		Tertile 3 vs. 1	
	β(95%CI)	p[2]	β(95%CI)	p[2]	β(95%CI)	p[2]	β(95%CI)	p[2]
Global Cognition								
Verbal reasoning	-0.27(-0.70,0.16)	0.318	-0.56(-0.95,-0.16)	0.024	-0.005(-0.35,0.34)	0.985	-0.69(-1.04,-0.34)	<0.001
Non-verbal reasoning	-0.28(-0.61,0.05)	0.318	-0.28(-0.58,0.03)	0.108	-0.03(-0.30,0.24)	0.985	-0.30(-0.57,-0.02)	0.035
Academics								
Word reading	-0.14(-0.38,0.09)	0.318	-0.20(-0.42,0.02)	0.108	-0.002(-0.19,0.19)	0.985	-0.30(-0.50,-0.11)	0.005
Mathematics	-0.10(-0.44,0.24)	0.564	-0.13(-0.44,0.19)	0.439	-0.09(-0.37,0.19)	0.985	-0.32(-0.61,-0.04)	0.035
Attention								
Sustained	0.04(-0.45,0.53)	0.871	-0.06(-0.51,0.39)	0.785	-0.23(-0.63,0.18)	0.919	-0.27(-0.68,0.14)	0.401
Variability	-0.16(-0.61,0.30)	0.782	-0.30(-0.72,0.12)	0.313	-0.10(-0.48,0.28)	0.919	-0.30(-0.68,0.09)	0.401
Commissions	0.40(-0.05,0.84)	0.322	0.39(-0.02,0.80)	0.236	0.02(-0.35,0.39)	0.919	0.07(-0.31,0.45)	0.836
Focused attention	0.10(-0.27,0.47)	0.782	0.05(-0.29,0.39)	0.785	0.04(-0.27,0.34)	0.919	-0.03(-0.34,0.28)	0.836
Processing Speed								
Visual-motor	0.14(-0.26,0.53)	0.498	-0.02(-0.39,0.35)	0.921	-0.17(-0.49,0.14)	0.562	-0.58(-0.90,-0.26)	0.001
Motor	-0.25(-0.69,0.19)	0.498	-0.23(-0.64,0.17)	0.528	0.05(-0.31,0.41)	0.783	-0.30(-0.66,0.07)	0.113
Memory								
Span	-0.28(-0.71,0.15)	0.555	-0.47(-0.87,-0.08)	0.075	-0.44(-0.79,-0.09)	0.051	-0.66(-1.01,-0.30)	0.001
New verbal encoding	0.18(-0.27,0.63)	0.581	0.12(-0.29,0.53)	0.626	0.12(-0.24,0.48)	0.686	-0.51(-0.88,-0.14)	0.012
Short-term verbal recall	0.25(-0.20,0.70)	0.555	0.22(-0.19,0.64)	0.583	0.04(-0.34,0.41)	0.854	-0.32(-0.69,0.06)	0.102
Long-term verbal recall	-0.01(-0.46,0.44)	0.974	0.10(-0.31,0.52)	0.626	0.13(-0.24,0.50)	0.686	-0.38(-0.75,-0.01)	0.063
Executive Function								
Perseveration	0.06(-0.47,0.59)	0.843	-0.21(-0.70,0.28)	0.545	-0.18(-0.63,0.26)	0.557	-0.30(-0.75,0.15)	0.198
Working memory	-0.45(-0.83,-0.06)	0.090	-0.42(-0.77,-0.06)	0.082	-0.21(-0.52,0.10)	0.375	-0.54(-0.86,-0.22)	0.003
Cognitive switching/flexibility	0.05(-0.44,0.54)	0.843	-0.05(-0.50,0.40)	0.839	0.08(-0.31,0.48)	0.677	-0.41(-0.81,-0.01)	0.063
Verbal fluency	0.07(-0.41,0.55)	0.843	-0.20(-0.64,0.25)	0.545	-0.35(-0.74,0.05)	0.344	-0.53(-0.93,-0.13)	0.018

[1]Models are adjusted for sex, diagnosis age, BMI, physical activity, smoking, and high-dose IV methotrexate, [2] p-values from linear regression models' t-tests, which are two sided, and adjusted for multiple comparisons using the false-discovery rate

Supplementary Table 12: Mean difference (95%CI) in neurocognitive z-score associated with the second or third tertile of PCPhenoAge EAA or mLTL residual compared to the first tertile among **survivors of Acute Lymphoblastic Leukemia (n=578)**.

	EAA[1]				mLTL[1]			
	Tertile 2 vs. 1		Tertile 3 vs. 1		Tertile 2 vs. 1		Tertile 3 vs. 1	
	β(95%CI)	p[2]	β(95%CI)	p[2]	β(95%CI)	p[2]	β(95%CI)	p[2]
Global Cognition								
Verbal reasoning	-0.17(-0.38,0.04)	0.157	-0.28(-0.50,-0.06)	0.028	0.24(0.02,0.47)	0.119	0.31(0.10,0.52)	0.017
Non-verbal reasoning	-0.09(-0.27,0.09)	0.342	-0.15(-0.34,0.05)	0.185	-0.06(-0.26,0.13)	0.520	0.002(-0.18,0.19)	0.985
Academics								
Word reading	-0.11(-0.25,0.02)	0.157	-0.17(-0.31,-0.04)	0.028	0.11(-0.03,0.25)	0.259	0.06(-0.07,0.19)	0.503
Mathematics	-0.17(-0.37,0.04)	0.157	-0.13(-0.35,0.08)	0.222	0.13(-0.08,0.34)	0.312	0.14(-0.07,0.34)	0.366
Attention								
Sustained	-0.12(-0.36,0.13)	0.727	-0.17(-0.43,0.10)	0.435	0.09(-0.18,0.35)	0.938	0.13(-0.12,0.38)	0.790
Variability	-0.12(-0.34,0.10)	0.727	-0.09(-0.33,0.15)	0.498	0.03(-0.21,0.26)	0.938	-0.03(-0.26,0.20)	0.790
Commissions	-0.07(-0.28,0.15)	0.742	-0.21(-0.44,0.03)	0.335	0.01(-0.22,0.24)	0.938	0.05(-0.18,0.27)	0.790
Focused attention	-0.00(-0.24,0.24)	0.978	-0.09(-0.34,0.17)	0.498	-0.07(-0.32,0.18)	0.938	-0.04(-0.28,0.20)	0.790
Processing Speed								
Visual-motor	-0.15(-0.34,0.04)	0.250	-0.07(-0.27,0.13)	0.848	0.001(-0.20,0.20)	0.992	0.12(-0.08,0.31)	0.242
Motor	-0.01(-0.25,0.22)	0.932	-0.02(-0.28,0.23)	0.848	-0.01(-0.25,0.24)	0.992	-0.16(-0.40,0.07)	0.242
Memory								
Span	-0.11(-0.30,0.08)	0.252	-0.05(-0.26,0.15)	0.609	0.10(-0.10,0.31)	0.981	0.05(-0.14,0.24)	0.833
New verbal encoding	-0.18(-0.41,0.06)	0.179	-0.22(-0.48,0.03)	0.219	-0.003(-0.25,0.25)	0.981	-0.02(-0.26,0.21)	0.846
Short-term verbal recall	-0.17(-0.39,0.05)	0.179	-0.13(-0.36,0.11)	0.393	-0.01(-0.25,0.22)	0.981	-0.08(-0.30,0.15)	0.833
Long-term verbal recall	-0.18(-0.41,0.05)	0.179	-0.20(-0.46,0.05)	0.219	-0.01(-0.25,0.24)	0.981	-0.06(-0.30,0.18)	0.833
Executive Function								
Perseveration	-0.14(-0.41,0.13)	0.585	-0.08(-0.37,0.21)	0.685	0.16(-0.13,0.44)	0.600	0.12(-0.16,0.39)	0.749
Working memory	-0.01(-0.18,0.17)	0.922	-0.04(-0.23,0.15)	0.685	0.06(-0.12,0.25)	0.600	0.05(-0.13,0.23)	0.749
Cognitive switching/flexibility	-0.23(-0.53,0.07)	0.501	-0.23(-0.55,0.09)	0.641	-0.08(-0.40,0.23)	0.600	0.05(-0.25,0.35)	0.749
Verbal fluency	-0.01(-0.22,0.20)	0.922	-0.10(-0.32,0.12)	0.685	0.12(-0.10,0.34)	0.600	0.12(-0.09,0.33)	0.749

[1]Models are adjusted for sex, diagnosis age, BMI, physical activity, smoking, cranial radiation dose, intrathecal methotrexate, high-dose IV methotrexate, and high-dose cytarabine [2] p-values from linear regression models' t-tests, which are two sided, and adjusted for multiple comparisons using the false-discovery rate

Supplementary Table 13: Mean difference (95%CI) in neurocognitive z-score associated with the third tertile of either DunedinPACE or PCGrimAge EAA compared to the first tertile among **survivors of Acute Lymphoblastic Leukemia (n=578)**.

	Dunedin PACE[1]				PCGrimAge EAA[1]			
	Tertile 2 vs. 1		Tertile 3 vs. 1		Tertile 2 vs. 1		Tertile 3 vs. 1	
	β(95%CI)	p[2]	β(95%CI)	p[2]	β(95%CI)	p[2]	β(95%CI)	p[2]
Global Cognition								
Verbal reasoning	-0.24(-0.45,-0.03)	0.109	-0.39(-0.63,-0.14)	0.005	-0.26(-0.47,-0.06)	0.049	-0.63(-0.87,-0.39)	<0.001
Non-verbal reasoning	-0.14(-0.33,0.04)	0.212	-0.28(-0.49,-0.06)	0.011	-0.14(-0.32,0.05)	0.216	-0.32(-0.53,-0.10)	0.005
Academics								
Word reading	-0.09(-0.23,0.04)	0.212	-0.22(-0.37,-0.07)	0.005	-0.09(-0.22,0.04)	0.216	-0.38(-0.53,-0.22)	<0.001
Mathematics	-0.13(-0.33,0.07)	0.216	-0.34(-0.57,-0.11)	0.005	-0.12(-0.32,0.08)	0.229	-0.34(-0.58,-0.10)	0.005
Attention								
Sustained	-0.09(-0.34,0.16)	0.697	-0.35(-0.64,-0.07)	0.031	-0.27(-0.51,-0.02)	0.067	-0.51(-0.80,-0.22)	0.001
Variability	-0.18(-0.40,0.05)	0.484	-0.27(-0.52,-0.01)	0.057	-0.20(-0.42,0.03)	0.084	-0.48(-0.74,-0.22)	0.001
Commissions	-0.01(-0.23,0.21)	0.931	-0.12(-0.37,0.13)	0.363	-0.20(-0.41,0.02)	0.084	-0.23(-0.49,0.02)	0.074
Focused attention	0.08(-0.16,0.32)	0.697	-0.42(-0.69,-0.14)	0.011	-0.29(-0.53,-0.05)	0.067	-0.43(-0.71,-0.15)	0.003
Processing Speed								
Visual-motor	-0.11(-0.30,0.08)	0.502	-0.39(-0.61,-0.17)	0.001	-0.24(-0.43,-0.05)	0.023	-0.49(-0.71,-0.27)	<0.001
Motor	0.02(-0.22,0.25)	0.885	-0.33(-0.60,-0.06)	0.015	-0.24(-0.48,-0.01)	0.041	-0.50(-0.77,-0.23)	<0.001
Memory								
Span	-0.10(-0.29,0.09)	0.292	-0.16(-0.38,0.06)	0.160	-0.16(-0.35,0.03)	0.125	-0.24(-0.47,-0.02)	0.031
New verbal encoding	-0.41(-0.64,-0.17)	0.001	-0.41(-0.67,-0.14)	0.006	-0.27(-0.50,-0.03)	0.071	-0.41(-0.69,-0.14)	0.006
Short-term verbal recall	-0.30(-0.52,-0.07)	0.012	-0.33(-0.58,-0.07)	0.015	-0.15(-0.37,0.07)	0.191	-0.30(-0.56,-0.04)	0.030
Long-term verbal recall	-0.47(-0.70,-0.24)	<0.001	-0.51(-0.77,-0.24)	0.001	-0.25(-0.48,-0.02)	0.071	-0.44(-0.71,-0.17)	0.006
Executive Function								
Perseveration	-0.12(-0.39,0.15)	0.618	-0.21(-0.53,0.10)	0.175	0.09(-0.18,0.36)	0.499	-0.40(-0.71,-0.08)	0.013
Working memory	-0.07(-0.24,0.11)	0.618	-0.19(-0.39,0.01)	0.092	-0.20(-0.37,-0.02)	0.070	-0.27(-0.47,-0.06)	0.013
Cognitive switching/flexibility	0.01(-0.28,0.31)	0.934	-0.60(-0.94,-0.26)	0.002	-0.25(-0.55,0.05)	0.141	-0.46(-0.80,-0.11)	0.013
Verbal fluency	-0.15(-0.36,0.06)	0.618	-0.26(-0.50,-0.02)	0.072	-0.22(-0.43,-0.02)	0.070	-0.39(-0.63,-0.14)	0.008

[1]Models are adjusted for sex, diagnosis age, BMI, physical activity, smoking, cranial radiation dose, intrathecal methotrexate, high-dose IV methotrexate, and high-dose cytarabine, [2] p-values from linear regression models' t-tests, which are two sided, and adjusted for multiple comparisons using the false-discovery rate

Supplementary Table 14: Mean difference (95%CI) in neurocognitive z-score associated with the third tertile of either DunedinPACE or PCGrimAge EAA compared to the first tertile among **survivors of Acute Lymphoblastic Leukemia, stratified by age at diagnosis (**Age <10 **n=446** Age ≥10 n=132**)**. Note stratified estimates were generated for models where the interaction term between EAA/mLTL and age at diagnosis was p<0.05.

	Dunedin PACE		PCGrimAge EAA		mLTL	
	β(95%CI)	p[1]	β(95%CI)	p[1]	β(95%CI)	p[1]
Processing Speed						
Visual-motor						
Age <10	-0.26 (-0.51, -0.02)	0.036	-0.35 (-0.59, -0.10)	0.005	0.08 (-0.14, 0.29)	0.503
Age ≥10	-0.82 (-1.24, -0.39)	<0.001	-1.04 (-1.47, -0.60)	<0.001	0.34 (-0.06, 0.74)	0.004
Executive Function						
Verbal Fluency						
Age <10					0.03 (-0.21, 0.27)	0.811
Age ≥10					0.50 (0.06, 0.94)	0.026

[1] Models are adjusted for sex, diagnosis age, BMI, smoking, physical activity, cranial radiation dose, high-dose IV methotrexate, IT methotrexate and high-dose cytarabine p-values from linear regression models' t-tests, which are two sided.

Supplemental Table 15: Statistically significant results from causal mediation analyses.

Treatment	EAA	Cognitive Outcome	*Total Effect*	*p-value*	*Natural Direct Effect*	*p-value*	*Natural Indirect Effect*	*p-value*	*Percentage Mediated*	*p-value*
CNS-Treated Survivors										
HD MTX	DunedinPACE	Verbal Reasoning	-0.28(-0.462,-0.099)	0.002	-0.219(-0.401,-0.038)	0.018	-0.061(-0.106,-0.015)	0.009	21.7(1.7,41.7)	0.034
HD MTX	DunedinPACE	Attention Variability	-0.285(-0.478,-0.092)	0.004	-0.228(-0.422,-0.035)	0.020	-0.057(-0.102,-0.012)	0.013	19.9(0.3,39.4)	0.046
Chest RT	PCGrimAge	Word Reading	0.251(0.055,0.448)	0.012	0.179(-0.009,0.367)	0.062	0.072(0.025,0.12)	0.003	28.8(4.2,53.4)	0.022
P-values from linear regression models’ t-tests within the causal mediation, which are two sided										

Supplemental Table 16: Overlap of differentially methylated CpG sites associated with treatment for childhood cancer and CpG sites included in epigenetic aging clocks.

**Treatment**	**CpG**	**HGNCgene**	**PCHorvath**	**PCHannum**	**PCPhenoAGE**	**DunedinPACE**	**PCGrimAge[1]**
Chest-RT	cg05316065	GSDMC	FALSE	FALSE	TRUE	FALSE	NA
Epipodophyllotoxins	cg06738602	PTGER2	TRUE	FALSE	FALSE	FALSE	NA
Alkylating agents	cg14200569	PRDM16	FALSE	FALSE	FALSE	TRUE	NA
Antimetabolites	cg14200569	PRDM16	FALSE	FALSE	FALSE	TRUE	NA
Asparaginase enzymes	cg14200569	PRDM16	FALSE	FALSE	FALSE	TRUE	NA
Epipodophyllotoxins	cg14200569	PRDM16	FALSE	FALSE	FALSE	TRUE	NA
Abdomen-RT	cg14200569	PRDM16	FALSE	FALSE	FALSE	TRUE	NA
Chest-RT	cg14200569	PRDM16	FALSE	FALSE	FALSE	TRUE	NA
Pelvic-RT	cg14200569	PRDM16	FALSE	FALSE	FALSE	TRUE	NA
Abdomen-RT	cg17061862	NA	FALSE	FALSE	FALSE	TRUE	NA
Abdomen-RT	cg19283806	CCDC102B	FALSE	TRUE	FALSE	FALSE	NA
Chest-RT	cg19283806	CCDC102B	FALSE	TRUE	FALSE	FALSE	NA
Pelvic-RT	cg19283806	CCDC102B	FALSE	TRUE	FALSE	FALSE	NA
Abdomen-RT	cg26581729	NPDC1	FALSE	FALSE	TRUE	FALSE	NA

[1] Catalog of CpG sites included on PC GrimAge is not currently publicly available.
