# Supplementary material for: Epigenetic age acceleration, telomere length, and neurocognitive function in long-term survivors of childhood cancer
Source: Nat Commun. 2025 Nov 27;16:10655. doi: 10.1038/s41467-025-65664-5 (PMC12660399; doi:10.1038/s41467-025-65664-5)
Supplement: Supplementary file 2 — Reporting Summary [file 41467_2025_65664_MOESM2_ESM.pdf]

nature portfolio

Corresponding author(s): AnnaLynn Williams and Kevin Krull

Last updated by author(s): 9-15-2025

Reporting Summary

Nature Portfolio wishes to improve the reproducibility of the work that we publish. This form provides structure for consistency and transparency in reporting. For further information on Nature Portfolio policies, see our Editorial Policies and the Editorial Policy Checklist.

Statistics

For all statistical analyses, confirm that the following items are present in the figure legend, table legend, main text, or Methods section.

n/a	Confirmed	
☐	☒	The exact sample size (*n*) for each experimental group/condition, given as a discrete number and unit of measurement
☐	☒	A statement on whether measurements were taken from distinct samples or whether the same sample was measured repeatedly
☐	☒	The statistical test(s) used AND whether they are one- or two-sided<br>*Only common tests should be described solely by name; describe more complex techniques in the Methods section.*
☐	☒	A description of all covariates tested
☐	☒	A description of any assumptions or corrections, such as tests of normality and adjustment for multiple comparisons
☐	☒	A full description of the statistical parameters including central tendency (e.g. means) or other basic estimates (e.g. regression coefficient) AND variation (e.g. standard deviation) or associated estimates of uncertainty (e.g. confidence intervals)
☐	☒	For null hypothesis testing, the test statistic (e.g. *F*, *t*, *r*) with confidence intervals, effect sizes, degrees of freedom and *P* value noted<br>*Give P values as exact values whenever suitable.*
☒	☐	For Bayesian analysis, information on the choice of priors and Markov chain Monte Carlo settings
☒	☐	For hierarchical and complex designs, identification of the appropriate level for tests and full reporting of outcomes
☒	☐	Estimates of effect sizes (e.g. Cohen's *d*, Pearson's *r*), indicating how they were calculated

Our web collection on statistics for biologists contains articles on many of the points above.

Software and code

Policy information about availability of computer code

Data collection	Epigenetic clocks were calculated using pyaging python package v0.1.22 (available at https://pyaging.readthedocs.io/en/latest/index.html)
Data analysis	All subsequent analyses were completed using SAS 9.4 (SAS Institute, Cary, N.C.), all the code underlying this manuscript can be found at https://doi.org/10.5281/zenodo.17127194.

For manuscripts utilizing custom algorithms or software that are central to the research but not yet described in published literature, software must be made available to editors and reviewers. We strongly encourage code deposition in a community repository (e.g. GitHub). See the Nature Portfolio guidelines for submitting code & software for further information.

Data

Policy information about availability of data

All manuscripts must include a data availability statement. This statement should provide the following information, where applicable:

- Accession codes, unique identifiers, or web links for publicly available datasets
- A description of any restrictions on data availability
- For clinical datasets or third party data, please ensure that the statement adheres to our policy

The dataset supporting the findings of this study has been deposited in Zenodo and is publicly available online at https://doi.org/10.5281/zenodo.17127194 as well as on the St. Jude Cloud at https://stjude.cloud. The DNA methylation data used in this study are accessible at the NCBI Gene Expression Omnibus website under accession no. GSE197674 for survivors and accession no. G S E 197676 for controls. 2. The whole-genome sequencing data is accessible through the St. Jude Cloud (https://stjude.cloud) under the accession number SJC-DS-1002.

Research involving human participants, their data, or biological material

Policy information about studies with human participants or human data. See also policy information about sex, gender (identity/presentation), and sexual orientation and race, ethnicity and racism.

Reporting on sex and gender	The term sex is used throughout the manuscript to refer to biological sex, which was self-reported. Information on gender was not asked of participants. The results are presented for both sexes combined and sex was considered a confounder and adjusted for in all analyses. For this study, we did not stratify by sex as we did not a priori hypothesize sex to be a modifier of the association between epigenetics and cognitive function.
Reporting on race, ethnicity, or other socially relevant groupings	Unfortunately, our analysis is restricted to survivors of European ancestry who all self-identified as non-Hispanic white. These were the only survivors with available data at the time of analysis. Future work is needed to explore modification by other ancestries.
Population characteristics	Our study includes data from 1413 long-term survivors of childhood cancer and 282 non-cancer controls recruited to the St. Jude Lifetime Cohort. Please refer to Table 1 for descriptive characteristics of this population.
Recruitment	Any patient who is treated at St. Jude Children's Research Hospital and has survived five or more years from diagnosis is eligible to participants in the St. Jude LIfetime Cohort (SJLIFE). Controls who are matched by age, sex, and race are recruited from the same geographic area of the patients. For this analysis we were restricted to a subsample of SJLIFE with available biospecimens and neurocognitive data. We compared demographic and clinical data between those included in this analysis and the remaining participants of SJLIFE and found no reasons to be concerned of potential selection biases. The larger SJLIFE cohort is representative of children with cancer treated at St. Jude and previous work has no demonstrated evidence of selection bias (PMCID : PMC3548083)
Ethics oversight	The SJLIFE protocol, biospecimen banking and genomic study were approved by the St. Jude institutional review board; participants provided written informed consent.

Note that full information on the approval of the study protocol must also be provided in the manuscript.

Field-specific reporting

Please select the one below that is the best fit for your research. If you are not sure, read the appropriate sections before making your selection.

☐ Life sciences ☒ Behavioural & social sciences ☐ Ecological, evolutionary & environmental sciences

For a reference copy of the document with all sections, see nature.com/documents/nr-reporting-summary-flat.pdf

Behavioural & social sciences study design

All studies must disclose on these points even when the disclosure is negative.

Study description	This is a cross-sectional quantitative study aimed at understanding the association between epigenetic modifications and neurocognitive functioning in long-term survivors of childhood cancer.
Research sample	This study employs pre-existing data from the St. Jude Lifetime Cohort (SJLIFE). Survivors treated for pediatric cancer at St. Jude Children's Research Hospital (SJCRH) were enrolled in the St. Jude Lifetime Cohort (SJLIFE); a prospective cohort established to characterize health outcomes among survivors of pediatric cancer; all participants provided informed consent. Age-, sex-, and race-matched non-cancer controls were recruited from the same geographic area of the survivors. Eligible survivors were diagnosed between 1962 and 2012 and survived ≥5 years from diagnosis, biospecimens and all other data were collected between 2008 and 2016 from the same SJLIFE visit where neurocognitive testing was completed. In our particular sample, the average age of survivors was 26 and 53% were male, remaining descriptives can be found in Table 1.
Sampling strategy	We used existing available data and biospecimens from participants of SJLIFE that were available as of March of 2016, when epigenetic assays were initiated. Therefore, this is a convenience sample of the larger cohort. No sample size calculations were performed. Our sample is similar in demographic and clinical characteristics to the larger SJLIFE cohort. Given we are using existing data, no participants opted not to participate in our study.
Data collection	Participants of SJLIFE complete an extensive neurocognitive assessment, clinical exam, questionnaire, and provide biospecimens. Because this study is not randomized, study staff and examiners are not blinded to the participants status as either a survivor of control. Please see Howell et al for an extensive description (PMID: 33374007) Specific to the neurocognitive exam, participants undergo a 2 hour in person neuropsychological assessment, for which the specific tests used are available in the supplement. Blood specimens were collected during the same time, processed and stored at -80 C for future processing.
Timing	There is only one time point for this study, biomarkers and cognitive function were measured at the same time point in long-term survivorship.
Data exclusions	As noted in Supplementary Figure 1, participants of SJLIFE who did not have biospecimen or neurocognitive data available were excluded from analyses. Survivors who were non-English speaking, had a genetic or neurodevelopmental syndrome associated with cognitive impairment but unrelated to cancer, or neurologic injury unrelated to cancer treatment (e.g. traumatic brain injury) were excluded. Survivors with CNS tumors were also excluded from the analyses because our prior work suggests the high doses of cranial

radiation used to treat these survivors overpowers genetic influences. Still when we compared those included in our analyses to those not, we saw no reasons for concerns over potential selection biases.

Non-participation	N/A
Randomization	This study is not randomized.

Reporting for specific materials, systems and methods

We require information from authors about some types of materials, experimental systems and methods used in many studies. Here, indicate whether each material, system or method listed is relevant to your study. If you are not sure if a list item applies to your research, read the appropriate section before selecting a response.

Materials & experimental systems

n/a	Involved in the study
☒	☐ Antibodies
☒	☐ Eukaryotic cell lines
☒	☐ Palaeontology and archaeology
☒	☐ Animals and other organisms
☒	☐ Clinical data
☒	☐ Dual use research of concern
☒	☐ Plants

Methods

n/a	Involved in the study
☒	☐ ChIP-seq
☒	☐ Flow cytometry
☒	☐ MRI-based neuroimaging

Plants

Seed stocks	*Report on the source of all seed stocks or other plant material used. If applicable, state the seed stock centre and catalogue number. If plant specimens were collected from the field, describe the collection location, date and sampling procedures.*
Novel plant genotypes	*Describe the methods by which all novel plant genotypes were produced. This includes those generated by transgenic approaches, gene editing, chemical/radiation-based mutagenesis and hybridization. For transgenic lines, describe the transformation method, the number of independent lines analyzed and the generation upon which experiments were performed. For gene-edited lines, describe the editor used, the endogenous sequence targeted for editing, the targeting guide RNA sequence (if applicable) and how the editor was applied.*
Authentication	*Describe any authentication procedures for each seed stock used or novel genotype generated. Describe any experiments used to assess the effect of a mutation and, where applicable, how potential secondary effects (e.g. second site T-DNA insertions, mosiacism, off-target gene editing) were examined.*
